# Coinfection of Malaria and Dengue Fever in Patients With Acute Undifferentiated Febrile Illnesses in Ghana: A Narrative Review

**DOI:** 10.1155/bmri/7174587

**Published:** 2026-07-27

**Authors:** Lordina Amissah, Evangeline Obodai, Godfred Amoah Appiah, Emmanuel Mensah Ayim, Petra Adjei, Senyo Botchie, Irene Ayi, Adwoa Asante-Poku, Charles Quaye, Stephen Osei-Wusu, Eric Kyei-Baafour, Justice Kumi, Joseph Humphrey Kofi Bonney

**Affiliations:** ^1^ Department of Virology, College of Health Sciences, Noguchi Memorial Institute for Medical Research, University of Ghana, Accra, Ghana, ug.edu.gh; ^2^ Department of Parasitology, College of Health Sciences, Noguchi Memorial Institute for Medical Research, University of Ghana, Accra, Ghana, ug.edu.gh; ^3^ Department of Bacteriology, College of Health Sciences, Noguchi Memorial Institute for Medical Research, University of Ghana, Accra, Ghana, ug.edu.gh; ^4^ Department of Immunology, College of Health Sciences, Noguchi Memorial Institute for Medical Research, University of Ghana, Accra, Ghana, ug.edu.gh; ^5^ Department of Clinical Pathology, College of Health Sciences, Noguchi Memorial Institute for Medical Research, University of Ghana, Accra, Ghana, ug.edu.gh

**Keywords:** acute undifferentiated febrile illnesses, coinfection, dengue fever, malaria

## Abstract

Malaria and dengue fever are coendemic vector‐borne diseases transmitted by *Anopheles* and *Aedes aegypti* mosquitoes, respectively, and are significant causes of acute undifferentiated febrile illness (AUFI) in Ghana. Evidence from Africa suggests an increasing prevalence of malaria‐dengue fever coinfection, and such coinfections may result in more severe clinical outcomes, though data specific to Ghana remain limited. This narrative review explores the prevalence, diagnostic challenges, and public health implications of malaria and dengue fever coinfections among AUFI patients in Ghana. This review highlights the critical need for integrated diagnostics, enhanced surveillance, and clinical awareness to improve patient outcomes and mitigate the dual burden of these coendemic vector‐borne diseases.

## 1. Introduction

Acute undifferentiated febrile illness (AUFI) is a common clinical and diagnostic challenge in tropical and subtropical regions, characterized by fever without specific organ‐related symptoms, making diagnosis challenging based on only clinical presentation. AUFI can be caused by various infectious and noninfectious conditions, including leptospirosis, rickettsiosis, dengue fever, malaria, typhoid fever, and inflammatory conditions [1–3].

Malaria and dengue fever are vector‐borne diseases with distinct transmission cycles involving specific mosquito vectors and causative agents. *Anopheles* mosquitoes transmit malaria, whereas *Aedes aegypti* mosquitoes transmit dengue fever [4]. The predominant causative agent of malaria in Ghana is *Plasmodium falciparum*, accounting for 98.1% of infections [4]. Conversely, dengue fever is caused by the dengue fever virus (DENV), a member of the Flaviviridae family, which has four distinct serotypes (DENV 1–4) [5]. Roughly 3.6 billion people living in tropical and subtropical zones are at risk for dengue fever and malaria infections. Dengue fever affects 50–200 million people globally annually, resulting in over 20,000 deaths [6]. In 2018, malaria cases were estimated at around 228 million worldwide, with a staggering 93% incidence in the African Region. In that same year, malaria claimed approximately 405,000 lives [7]. The coexistence of *Anopheles* and *Aedes* vectors and a conducive ecological niche presents unique challenges for public health in Ghana. Several studies have reported an increasing prevalence of malaria and dengue fever coinfection in Africa. For instance, a systematic review by Gebremariam et al. revealed that the prevalence of malaria and acute dengue fever coinfection rose significantly from 0.9% in 2008–2013 to 5.5% in 2018–2021 [8]. Similarly, another study found a coinfection prevalence of 6.6% (*n* = 26/395) among febrile patients [9].

Coinfection of malaria and dengue fever could result in more severe clinical outcomes, including a higher risk of deep bleeding, hepatomegaly, and jaundice, in comparison to mono‐infections [10, 11]. This is concerning, considering that the clinical overlap between malaria and dengue fever might lead to misdiagnosis, potentially delaying effective therapy [12]. Furthermore, in Ghana, as in other low‐resource settings in sub‐Saharan Africa, arboviruses like DENV are not routinely tested for, and clinicians may consider them only as a secondary differential when malaria has been excluded [13]. Given the overlapping clinical features of malaria and dengue fever, the increasing prevalence of coinfections, and the diagnostic challenges in resource‐limited settings like Ghana, it is imperative to better understand the burden and characteristics of these dual infections. This narrative review, therefore, explores malaria and dengue fever coinfection among patients with AUFIs in Ghana, drawing attention to the epidemiological patterns, diagnostic challenges, and public health implications.

### 1.1. Epidemiology of Malaria and Dengue Fever

Malaria constitutes 2.6% of the entire global disease burden [14]; Africa bears the highest burden in terms of both malaria cases and fatalities [15]. Although incidence rates have remained stable in recent years, the number of cases has increased due to a growing population being at risk. Between 2000 and 2023, malaria incidence and mortality rates declined by 36% and 63%, respectively [16].

In Ghana, malaria remains a major public health issue and the leading cause of morbidity and mortality, particularly among vulnerable populations such as children under 5 years of age and pregnant women. In 2020, the country had more than 5.2 million confirmed cases of malaria and 308 deaths linked to malaria [17]. In the same year, malaria was responsible for 26.1% of outpatient department (OPD) attendance, 32.5% of hospital admissions, and 0.37% of all fatalities [18]. Dengue fever, though less studied than malaria in Ghana, is an emerging threat [19]. Until 2024, Ghana had only reported sporadic cases or serological evidence of dengue fever [20, 21]. In 2024, a widespread outbreak of dengue fever resulted in over 1400 suspected cases and 206 confirmed cases [22]. The actual burden of dengue fever in Ghana is likely higher due to underreporting, misdiagnosis (often as malaria), and limited diagnostic capacities [13]. This challenge is further compounded by the lack of accurate point‐of‐care tests or the sole reliance on microscopy for malaria diagnosis, which has shown variable accuracy across different clinical sites [10].

## 2. Clinical Presentation and Current Diagnostics

**Figure 1:**
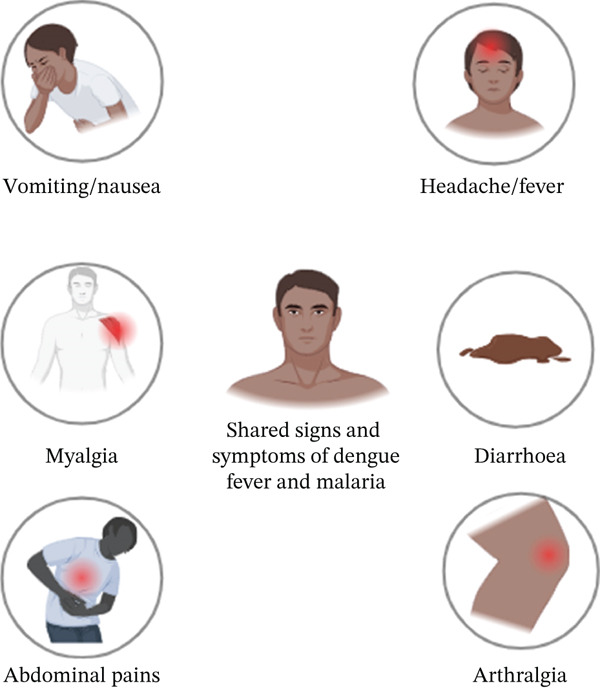
Overlapping clinical presentation of dengue fever and malaria. The image was created using the BioRender tool.

Dengue fever and malaria share several overlapping clinical features: fever, headache, myalgia, arthralgia, rash, nausea, vomiting, and abdominal pain (Figure 1) [23]. Dengue classically progresses through febrile, critical, and recovery stages, though these are not always well‐defined in practice. The febrile phase mirrors malaria closely, but as the disease advances, a subset of patients enter a critical phase marked by increased vascular permeability, thrombocytopenia, and other warning signs, including persistent vomiting, abdominal pain, and mucosal bleeding [24]. In those who further deteriorate, clinical presentations diverge into severe dengue marked by severe hemorrhage, severe plasma leakage that may lead to respiratory shock, pulmonary fluid accumulation with respiratory distress, or severe multiorgan involvement [24]. Similarly, malaria can progress into severe malaria characterized by multiple organ involvement, including neurological (multiple convulsions), pulmonary (acute respiratory distress syndrome, pulmonary oedema), hepatic (bilirubin > 3.0 mg/dL, elevated transaminases), hypoglycaemia (< 40 mg/dL), and renal (creatinine > 3.0 mg/dL) involvement [25]. In coinfections, however, the likelihood of a higher risk of severe disease, tachycardia, deep bleeding, hepatomegaly, nausea, vomiting, and jaundice increases [26].

Laboratory diagnostic methods for malaria and dengue fever include microscopy (specifically for malaria), serology (rapid diagnostic tests [RDTs], enzyme‐linked immunosorbent assay [ELISA]), and molecular techniques like PCR, each with its advantages and limitations (Table 1). Microscopy remains the gold standard for malaria diagnosis, offering high specificity and sensitivity when performed by skilled technicians [27]. However, it is labor‐intensive, time‐consuming, and requires significant expertise, which can lead to therapeutic delays [28]. RDTs have become a primary diagnostic tool in many endemic areas due to their quick turnaround time, simplicity, and ability to detect *P. falciparum* and nonfalciparum infections [27]. The primary target antigen of RDTs is the histidine‐rich protein 2 (*Pf-HRP2*). Alternative targets include aldolase and *P. falciparum*‐specific or pan‐parasite lactate dehydrogenase (*Pf-pLDH, pLDH*) [30]. However, RDTs have varying sensitivities and specificities depending on the target antigen, malaria prevalence, and temperature [31]. False positives can occur due to persistent parasite antigens posttreatment [35]. Additionally, false negatives can also arise from very low or high parasitemia, malfunctioning test kits, or *P. falciparum* species with variations or absence of *HRP2* proteins [30]. Molecular methods like PCR offer higher sensitivity and specificity compared with microscopy and RDTs, especially in low‐transmission areas or for detecting mixed infections [32]. Schlabe et al. reported the use of PCR to diagnose an RDT‐negative malaria infection by a *P. falciparum* strain with mutations/deletions in the histidine‐rich protein genes [30]. However, PCR is more expensive, requires specialized equipment and trained personnel, limiting its utility in resource‐limited settings [28]. In cases of coinfection, exclusive reliance on malaria‐biased RDTs presents a risk of overlooking dengue fever. This is concerning, as studies have shown that nonmalarial aetiologies cause a significant proportion of fevers in malaria‐positive children [35].

**Table 1:** Summary of the advantages, disadvantages, and detection windows of malaria and dengue fever diagnostics.

Diagnostic method	Advantages	Disadvantages	Detection window	Sources
Malaria				
Microscopy (thick and thin blood films)	• High specificity	• Labor‐intensive	Parasitemic phase: most reliable when parasitaemia is microscopically detectable	[27–29]
	• High sensitivity	• Time‐consuming		
	• Enables species identification and parasite density quantification	• Requires a high level of expertise		
Malaria rapid RDT (HRP‐2/pLDH antigen detection)	• Fast and simple	• False positives due to HRP‐2 antigen persistence posttreatment	Day 0 onward, HRP‐2 antigen may persist for weeks after treatment, producing false‐positive results	[27, 28, 30, 31]
	• No specialized equipment required			
	• Suitable for peripheral and resource‐limited settings	• False negatives at low parasitaemia		
		• Cannot quantify parasite density		
PCR	• High sensitivity	• Highly expensive	Parasitaemic phase; detects very low‐level infections below the threshold of microscopy	[28, 32]
	• High specificity	• Requires specialized equipment		
	• Detects submicroscopic parasitaemia missed by microscopy and RDT	• Requires trained personnel		
	• Enables species differentiation			
Dengue fever				
RDT (NS1 antigen)	• Rapid point‐of‐care result	• Reduced sensitivity in secondary infection	Days 0–9 post‐onset; optimal sensitivity Days 0–4 (peak viremia)	[33, 34]
	• Detects dengue early before antibody response develops	• Performance varies by serotype and DPO		
		• Potential cross‐reactivity with other flaviviruses		
ELISA (NS1 antigen)	• Higher sensitivity and specificity than NS1 RDT	• Requires laboratory infrastructure	Days 1–9 post‐onset; remains useful beyond Day 4 when viraemia subsides	[33, 34]
	• Serotype‐specific variants available	• More expensive than RDT		
		• Potential cross‐reactivity with other flaviviruses		
ELISA (IgM or IgG antibodies)	More sensitive than IgM RDT	• Poor sensitivity in the early acute phase	From Day 4–5 post‐onset; peaks approximately 2 weeks after symptom onset; may persist up to 3 months	[33, 34]
		• Flavivirus cross‐reactivity		
PCR	• Highest sensitivity in early infection	• Highly expensive	Days 0–5 post‐onset; highest sensitivity during peak viraemia (0–4 DPO)	[33, 34]
	• Enables DENV serotyping	• Requires specialized equipment and trained personnel		
	• Gold standard for early‐phase molecular confirmation	• Sensitivity varies considerably across studies and settings		

Abbreviations: DENV, dengue virus; DPO, days post‐onset of symptoms; ELISA, enzyme‐linked immunosorbent assay; HRP‐2, histidine‐rich protein 2; NS1, nonstructural protein 1; PCR, polymerase chain reaction; pLDH, pan‐parasite lactate dehydrogenase.

Diagnosing dengue fever relies on the clinical setting and the timing of the test relative to the days post‐onset of symptoms (DPO). Positive cases are confirmed by viral detection (RT‐PCR or viral isolation) or by specific antibody assays. RT‐PCR is sensitive during the early viraemic phase (0–4 DPO), though its broader utility is often limited by high costs and a narrow detection window. Alternatively, the NS1 glycoprotein, expressed across all DENV variants, remains detectable for up to 10 DPO [33]. Antidengue IgM antibodies typically emerge around 4 DPO and persist for months. Nonetheless, a negative antidengue IgM result cannot definitively rule out dengue infection unless combined with RT‐PCR or NS1 testing [34, 36]. Similarly, a positive antidengue IgG is an unreliable standalone marker due to lifelong persistence from frequent vector exposure and cross‐reactivity with other flaviviruses in endemic areas [36]. Robust polyclonal B‐cell activation previously described in malaria infection has been speculated to cause false‐positive results in dengue IgM and IgG assays in malaria endemic settings [37]. A recent systematic review and meta‐analysis by Pillay et al. revealed RT‐PCR demonstrated the highest sensitivity at 0–4 DPO, followed by NS1 ELISA (sensitivity, 90%; specificity, 93%), beyond 4 DPO. Antidengue IgM ELISA performed poorly in the early acute phase, with a sensitivity of only 17% at 0–4 days, rising to 71% at 1–7 days [33]. Recent methodologies have utilized serum concentration via ultrafiltration to significantly enhance dengue RDT performance [38].

It is evident that a multifaceted diagnostic approach is necessary to improve patients′ outcomes. Additionally, healthcare providers should be made aware of the possibility of coinfections in AUFIs and investigate accordingly [39].

### 2.1. Environmental Factors and Vector Cotransmission

Rainfall patterns fundamentally shape vector breeding, as both mosquito species require standing water to complete their life cycles, although with different preferences [40]. The *Anopheles* spp. breed in clean, sun‐exposed water bodies created by seasonal rainfall, whereas *A. aegypti* preferentially breeds in smaller artificial containers [41]. The increasing unpredictability of rainfall patterns in Ghana, characterized by more intense precipitation events followed by dry periods, has created the ideal conditions for both vectors to thrive simultaneously in periurban environments where natural and artificial breeding sites coexist [42].

Temperature is equally crucial in vector biology and disease transmission dynamics; temperatures between 25°C–30°C represent an optimal range for *Anopheles* and *Aedes* mosquitoes, accelerating their development cycles and increasing biting frequencies [43]. Studies indicate that nighttime temperature increases have benefited *Anopheles* mosquitoes, whereas daytime warming favors *Aedes* mosquitoes′ activity, likely to create a 24‐h transmission cycle [44].

Climate change further amplifies these environmental factors through several mechanisms. Extended dry seasons drive increased external domestic water storage practices favorable to *Aedes* mosquito breeding, whereas more intense but less predictable rainfall creates temporary *Anopheles* mosquito breeding sites in unusual locations and times [45]. Such climatological shifts may disrupt traditional seasonal transmission patterns, with modeling studies suggesting the potential for more year‐round transmission in regions that previously exhibited distinct seasonal disease profiles [43]. Environmental factors interact with human behavior and ecological change, creating complex feedback loops that enhance the cotransmission potential [46].

### 2.2. Malaria‐Dengue Fever Coinfection and Public Health Implications in Ghana

Although a systematic review of African data indicates a pooled coinfection prevalence of 4.2% (with West Africa notably lower at 1.6%) [8], localized findings suggest the prevalence of dengue fever and malaria coinfection in Ghana remains largely under‐investigated. Stoler et al. reported dengue seropositivity rates (IgM and IgG) of 3.2% (*n* = 7/218) and 21.6% (47/218), respectively, among malaria parasite‐positive children, across the coastal savannah, tropical‐forested transition, and dry southern savannah zones. Despite these findings, the study did not detect DENV RNA or rule out the potential for flavivirus cross‐reactivity [47]. More recently, Amoako et al. detected DENV‐2 in a patient who was also positive for malaria (*P. falciparum* detected) using a multipathogen, real‐time PCR‐based assay [48].

As noted by Stoler and Awandare, up to a third of febrile patients in sub‐Saharan Africa may not receive a correct diagnosis, highlighting the importance of considering multiple aetiologies in cases of AUFIs [49]. It is plausible that the few malaria‐dengue fever coinfections in Ghana may be the tip of the proverbial iceberg, and there is a clear need for improved surveillance and diagnostic guidelines to accurately and promptly determine coinfection rates. Stoler and Awandare have argued that, in sub‐Saharan Africa more broadly, malaria has become the default diagnosis for febrile patients in low‐resource settings, inadvertently creating a “malaria‐industrial complex”. They suggest this institutionalization has historically contributed to systemic bias, where other febrile diseases like dengue fever are sidelined [49]. Such diagnostic bias has been associated elsewhere with inappropriate treatment, increased morbidity and mortality, and the potential for drug resistance [2].

The management of malaria‐dengue fever coinfection can be challenging, given that the two infections carry divergent pathophysiological risks that can be exacerbated by undifferentiated treatment [26]. For antipyresis, paracetamol (acetaminophen) is the only agent recommended in both conditions and therefore represents the sole safe choice when coinfection is suspected or confirmed. However, it carries a cumulative hepatotoxic risk when coadministered with other potentially hepatotoxic agents. Ibuprofen, aspirin, and other nonsteroidal anti‐inflammatory drugs (NSAIDs) are explicitly contraindicated in both diseases [50, 51]. Malaria and dengue fever demand fundamentally distinct fluid management approaches that can be clinically difficult to reconcile in coinfection. In uncomplicated dengue without warning signs, oral rehydration is preferred, using oral rehydration solution (ORS), fruit juice, or electrolyte‐containing fluids to compensate for losses from fever and vomiting; intravenous (IV) fluids (0.9% saline or Ringer′s lactate) are reserved for patients who cannot tolerate oral intake. When dengue warning signs are present, IV isotonic crystalloid is commenced at 5–7 mL/kg/h, titrated down over 24–48 h based on hematocrit and clinical response to maintain minimum volume necessary for perfusion and a urine output of approximately 0.5 mL/kg/h; fluid overload during or after the critical phase carries a significant risk of pulmonary oedema and congestive heart failure. In severe dengue with shock, more aggressive resuscitation with crystalloid boluses is required, guided by serial hematocrit measurements and haemodynamic monitoring [51]. In malaria, by contrast, fluid requirements must be assessed individually, as adults with severe malaria are highly vulnerable to fluid overload, whereas children are more likely to be dehydrated; rapid bolus infusion of colloid or crystalloids is contraindicated in severe malaria, and there is insufficient data to support generalized fluid replacement protocols [50].

To address these issues, there is a need for increased awareness among healthcare workers and implementation of a socio‐environmental approach for the diagnosis and management of acute febrile illness etiology in Ghana. Vaccination programs could complement other control measures, such as vector control, providing long‐term immunity and lowering the burden on healthcare systems [52]. Currently, Ghana, under the Malaria Vaccine Implementation Programme (MVIP), has rolled out the World Health Organization′s approved malaria vaccine in seven administrative regions to reduce the disease burden [53]. However, no dengue fever vaccine has been rolled out in Ghana yet.

## 3. Research Gaps and Future Outlook

Although the notable gap in malaria‐dengue fever coinfection studies in Ghana limits our understanding of the true prevalence and impact of coinfection in the country, healthcare workers must also recognize that malaria and dengue fever can coexist in endemic settings and determine the most appropriate diagnostic algorithm for AUFIs. As noted by Stoler and Awandare, empirical treatment of AUFIs could miss dengue fever cases in coinfections [49]. As a result, development and validation of multiplex diagnostics capable of simultaneously detecting malaria and DENV antigens or antibodies may improve detection of coinfections in Ghana, though their feasibility and performance would need to be established. Community engagement and social mobilization could also support efforts to raise awareness of malaria‐dengue fever coinfections in Ghana, covering symptoms, prevention, and the importance of seeking timely diagnosis and treatment.

## 4. Conclusion

The available Ghanaian data suggest that malaria‐dengue fever coinfections carry significant public health implications, though the limited number of studies identified in this review precludes firm conclusions about the true prevalence or clinical impact of coinfection in this setting. Nonetheless, existing evidence indicates that diagnostic gaps likely obscure the actual burden of coinfections and may place additional pressure on an already strained healthcare system in Ghana. Addressing these gaps will require a multipronged approach that includes improving diagnostic capabilities across all healthcare levels in Ghana, enhanced surveillance, and expanded research efforts specifically tailored to the Ghanaian context. Recognition of the potential for coinfection among clinicians working in Ghana, together with appropriate diagnostic algorithms and health policy adaptations, could contribute to improved patient outcomes and a more accurate characterization of the febrile illness burden in the country.

**Nomenclature**
AUFI: Acute undifferentiated febrile illness
DENV: Dengue fever virus
DHF: Dengue hemorrhagic fever
DPO: Days post‐onset of symptoms
DSS: Dengue shock syndrome
ELISA: Enzyme‐linked immunosorbent assay
IgG: Immunoglobulin G
IgM: Immunoglobulin M
IV: Intravenous
MVIP: Malaria Vaccine Implementation Programme
NS1: Nonstructural protein 1
NSAIDs: Nonsteroidal anti‐inflammatory drugs
OPD: Outpatient department
ORS: Oral rehydration solution
PCR: Polymerase chain reaction
*Pf-HRP2*
: *Plasmodium falciparum* histidine‐rich protein 2
*Pf-pLDH*
: *Plasmodium falciparum*‐specific lactate dehydrogenase
*pLDH*
: Pan‐parasite lactate dehydrogenase
RDT: Rapid diagnostic test
RNA: Ribonucleic acid
RT‐PCR: Reverse transcription polymerase chain reaction
WHO: World Health Organization

## Author Contributions

L.A. and G.A.A. conceived the original idea of this review with inputs from J.H.K.B.; L.A., G.A.A., E.M.A., P.A., and S.B. prepared the original draft, which was reviewed and edited by L.A., G.A.A., E.O., I.A., A.A‐P., C.Q., S.O‐W., E.K‐B., J.K., and J.H.K.B.; J.H.K.B. provided oversight and leadership.

## Funding

This work was funded by the West African Health Organization (WAHO), the specialized health institution of the Economic Community of West African States (ECOWAS), under grant/project reference profiling malaria and other febrile illnesses to improve malaria case detection and management in West Africa—PROMAFILD/Project.

## Disclosure

All authors read and approved the final manuscript.

## Conflicts of Interest

The authors declare no conflicts of interest.

## Data Availability

No new data generated.
